# Reliable blood cancer cells' telomere length evaluation by qPCR

**DOI:** 10.1002/cam4.2816

**Published:** 2020-03-06

**Authors:** Joana Ropio, Alain Chebly, Jacky Ferrer, Martina Prochazkova‐Carlotti, Yamina Idrissi, Lamia Azzi‐Martin, David Cappellen, Anne Pham‐Ledard, Paula Soares, Jean‐Philippe Merlio, Edith Chevret

**Affiliations:** ^1^ Bordeaux University INSERM U1053 Bordeaux Research in Translational Oncology (BaRITOn) Cutaneous Lymphoma Oncogenesis Team Bordeaux France; ^2^ Porto University Institute of Biomedical Sciences of Abel Salazar Porto Portugal; ^3^ Instituto de Investigação e Inovação em Saúde Porto Portugal; ^4^ Institute of Molecular Pathology and Immunology University of Porto (Ipatimup) Cancer Biology group Porto Portugal; ^5^ Faculty of Medicine Medical Genetics Unit Saint Joseph University Beirut Lebanon; ^6^ Bordeaux University UFR des Sciences Médicales INSERM U1053 Bordeaux Research in Translational Oncology (BaRITOn) Bordeaux France; ^7^ Bordeaux University Hospital Center Tumor Bank and Tumor Biology Laboratory Pessac France; ^8^ Bordeaux University Hospital Center Dermatology Department Bordeaux France; ^9^ Department of Pathology Faculty of Medicine University of Porto Porto Portugal

**Keywords:** cancer, qPCR, southern blot, telomere length, tumor

## Abstract

**Background:**

Telomere shortening is linked to a range of different human diseases, hence reliable measurement methods are needed to uncover such associations. Among the plethora of telomere length measurement methods, qPCR is reported as easy to conduct and a cost‐effective approach to study samples with low DNA amounts.

**Methods:**

Cancer cells’ telomere length was evaluated by relative and absolute qPCR methods.

**Results:**

Robust and reproducible telomere length measurements were optimized taking into account a careful reference gene selection and by knowing the cancer cells ploidy. qPCR data were compared to “gold standard” measurement from terminal restriction fragment (TRF).

**Conclusions:**

Our study provides guidance and recommendations for accurate telomere length measurement by qPCR in cancer cells, taking advantage of our expertise in telomere homeostasis investigation in primary cutaneous T‐cell lymphomas. Furthermore, our data emphasize the requirement of samples with both, high DNA quality and high tumor cells representation.

## Graphical abstract

In this original article we aimed to validate the applicability of qPCR when assessing telomere length in cancer cells, taking advantage of our expertise on telomere homeostasis investigation in primary cutaneous T‐cell lymphomas

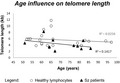

## 1 INTRODUCTION

Telomeres are highly conserved repetitive (TTAGGG)n DNA‐protein structures located at the ends of eukaryotic chromosomes.^1^, ^2^ They have important functions in chromosomal stability and replication.^3^ Due to the “end replication problem” telomeric sequences shorten after every cell division, leading to replicative senescence, cell cycle arrest, or apoptosis.^4^, ^5^ Telomere progressive shortening can potentially induce genetic instability and neoplastic transformation and may be counteracted by telomerase, an enzyme specialized in the elongation of telomeric ends.^6^ This enzyme is silenced in most somatic cells and expressed in about 90% of cancer cells.^7^ The remaining 10% of cancers activate an alternative telomere length mechanism known as ALT.^8^ The reexpression of telomerase allows cells to circumvent senescence and to achieve immortalization by maintaining functional telomeres.^9^ As protectors of chromosome ends, telomeres are involved in the pathogenesis and clinical progression of human diseases, including cancer and a number of metabolic and inflammatory diseases.^10^, ^11^, ^12^ Considering the role of telomere length in biological homeostasis, there has been a growing interest in measuring telomere length accurately and efficiently.^13^, ^14^

A wide range of methods have been developed to measure telomere length, such as terminal restriction fragment (TRF) analysis by Southern blot, quantitative polymerase chain reaction (qPCR) amplification of telomere repeats relative to a single copy gene, and fluorescent in situ hybridization (FISH) to quantify telomere repeats in individual cells (interphase‐FISH and flow‐FISH) or in individual arm chromosome (metaphase‐FISH). The advantages and drawbacks of each method have been discussed in many reviews.^15^, ^16^, ^17^, ^18^, ^19^ TRF analysis was the first technique developed for telomere length measurement, and is often considered as the “gold standard” for all other techniques. In this procedure, genomic DNA is exhaustively digested by a cocktail of restriction enzymes, resulting in short genomic fragments and longer uncut telomeres. Telomere fragments are then resolved by agarose gel electrophoresis and detected by Southern blot using a labeled telomere probe. The average telomere length is determined by quantification of the intensity of labeled telomere DNA smear, compared to a DNA ladder with known fragment sizes in kilobases (kb). TRF analysis requires large amounts of DNA (0.5 to 10 μg) and has a maximum detection threshold of around 20 kb because of the resolutive nature of agarose gel electrophoresis.^20^, ^21^ Nowadays, qPCR is the most commonly used method for assessing telomere length. qPCR is low cost, not very time consuming, is amenable to a high‐throughput format and, unlike TRF assay, it can be performed with small quantities of DNA (less than 100 ng).^22^, ^23^ In this procedure, telomere length is quantified by comparing the amplification of the telomere product (T) to the amplification of a single copy gene (S). The T/S ratio yields a value that is proportional to average telomere length, allowing the determination of relative telomere length.^24^, ^25^, ^26^, ^27^ Nevertheless, to obtain accurate, precise, and reproducible data, several factors should be considered.^28^, ^29^

One of the main hurdles when studying cancer cells is the scarce biological material recovered which constraints molecular biology analysis. Thus, qPCR approaches present a substantial advantageous tool for cancer cells’ telomere length evaluation. In this work, we aimed to compare and validate the applicability of qPCR when assessing telomere length in cancer cells, taking advantage of our expertise in telomere homeostasis investigation in primary cutaneous T‐cell lymphomas (CTCL). CTCL are a heterogeneous group of lymphoproliferations including entities with indolent, intermediate, and aggressive clinical behavior, in which we previously reported that telomere shortening was associated with disease aggressiveness.^30^

## 2 MATERIAL AND METHODS

### 2.1 Cell lines

Five CTCL cell lines were analyzed in this study. Three cutaneous anaplastic large cell lymphoma (c‐ALCL): Mac1, Mac2A, and Mac2B 31 (DSMZ), one transformed mycosis fungoïdes (T‐MF): MyLa 2973,^32^ kindly provided by Dr K. Kaltoft (Aarhus, Denmark) and one Sézary syndrome (Sz): HuT78 33 (ATCC). They were cultured as suspension cells in Roswell Park Memorial Institute Medium (RPMI) 1640 media (Gibco) supplemented with 100U/mL penicillin, 100µg/mL streptomycin (Gibco) and 10% fetal bovine serum (Eurobio), except for HuT78 cells, which were supplemented with 20% fetal bovine serum. All cell lines were maintained at 37ºC with 5% CO2 and regularly tested for mycoplasma contamination.

### 2.2 Patients and healthy donors

Sz patients (n = 10, 51 ≤ age ≤86, mean age 71), were selected from the dermatology department of University Hospital Center (CHU) of Bordeaux, diagnosed according to the criteria of the World Health Organization and the European Organization for Research and Treatment of Cancer (WHO‐EORTC).^34^ Healthy donors (n = 21, 52 ≤ age ≤97, mean age 68) were recruited from both Etablissement Français du Sang (EFS), and CHU of Bordeaux, France. Peripheral blood mononuclear cells from Sz patients and healthy donors were isolated by PANCOLL® density gradient centrifugation (PAN‐Biotech). Each patient gave a written consent.

### 2.3 Conventional cytogenetics

MyLa, HuT78, Mac1, Mac2A, and Mac2B cells in the logarithmic growth phase were incubated with Colcemid (Gibco). Cells were harvested and fixed according to the standard cytogenetic methods (KCl hypotonic treatment and ethanol‐acetic acid fix Normapur 3:1 ratio). Fixed cells were spread on Superfrost glass slides (Thermo Scientific). Metaphases were treated for R‐banding and then scanned on AxioImager Z1 (Zeiss) using Metafer software (MetaSystems). For each cell line, 5 to 10 metaphases were analyzed using Ikaros karyotyping software (Metasystems). Karyotypes were assessed by a cytogeneticist and chromosomal formulas were written according to International System for Human Cytogenetic (ISCN) 2016 nomenclature.

### 2.4 Multicolor Fluorescence in situ Hybridization (mFISH)

mFISH karyotype was carried out in accordance with supplier's instructions using 24XCyte kit (MetaSystems) on cell lines and patient metaphase cells spreads. Cytogenetic preparations were performed as previously described.^35^ For each sample, nearly 20 metaphases were analyzed by means of ISIS software for mFISH (MetaSystems). Chromosome abnormalities were defined according to ISCN 2016 recommendations.

### 2.5 DNA Extraction

Genomic DNA was extracted, by a salt precipitation method adapted from Roylance *et al*.^36^ Briefly, about 3 to 5x10^6^ cells were washed with PBS. The pellets were resolved in nuclei lysis buffer (10 mM Tri‐HCl/pH 8.2, 2 mM EDTA, 400 mM NaCl) completed with 0.1% Nonidet P‐40, 1/10 RNAse A (10mg/ml) and proteinase K buffer solution (2mg/ml proteinase K, 2mM EDTA, 1% SDS), prepared freshly prior to use. Suspensions were incubated overnight at 43°C. The DNA was precipitated with ethanol and then resolved in DNase‐RNase free distilled water. DNA concentration was measured by Nanodrop 2000 spectrophotometer (Thermo Fisher Scientific) and its quality was further analyzed by classic agarose gel electrophoresis. The extracted material was maintained at 4ºC during quality assessment and qPCR analysis, otherwise it was stored at −20°C.

### 2.6 Terminal Restriction Fragment telomere length measurement

Telomere measurement was carried out following the protocol of TeloTAGGG Telomere Length Assay Kit (Roche). Briefly, 1.5 µg of DNA was digested with Hinfl and RsaI enzymes. Digested samples were run on agarose gel and the telomere fragments were then transferred to a nylon membrane Hybond‐N+ (Amersham). DNA was fixed and a DIG‐labeled telomeric probe was hybridized to the membrane. After a series of stringent washes and incubation with the secondary anti‐DIG antibody, the telomeric DNA was detected by chemiluminescent imaging (ImageQuant LAS 4010, GE Healthcare). Images were analyzed using ImageJ software (IJ 1.46r). Telomere content was calculated by the equation: TRF mean = ΣOD*i*/Σ(OD*i*/*Li*), where OD*i* is the chemiluminescent signal and *Li* is the length of the TRF fragment at position *i*.

### 2.7 qPCR relative telomere length measurement

Telomere length was calculated by a standard quantitative qPCR assay as previously reported.^30^ The normalizing control gene used was Kallikrein Related Peptidase 3 (KLK3), located at 19q13.33. Fifty nanograms of target DNA was added to a reaction containing the pair of primers (telomere or KLK3) and Takyon^TM^ No Rox SYBR® MasterMix dTTP Blue (Eurogentec), in a total reaction volume of 25µl, according to the manufacturer's instructions. PCR experiments were carried out on a Stratagene Mx3005P system (Agilent Technologies) and analyzed with MxPro 4.01 QPCR software Stratagene (Agilent Technologies).

Primer sequences for both telomeres and KLK3 were as follows:

Telc 5'‐TGTTAGGTATCCCTATCCCTATCCCTATCCCTATCCCTATCCCTAACA‐3'.

Telg 5'‐ACACTAAGGTTTGGGTTTGGGTTTGGGTTTGGGTTAGTGT‐3'.^24^

KLK3‐forward 5'‐AGGCTGGGGCAGCATTGAAC‐3'.

KLK3‐reverse 5'‐CACCTTCTGAGGGTGAACTTG‐3'.

Telomere (2 cycles of 95°C for 20 sec and 49°C for 20 sec, followed by 30 cycles of 95°C for 20 sec and 60°C for 20 sec, with signal acquisition) and KLK3 (40 cycles of 95°C for 20 sec and 60°C for 20 sec, with signal acquisition) reactions were run in separate 96‐well plates.

Data were collected from triplicate reactions for each sample (cell lines, patients, and healthy donors). Triplicate values were accepted when the standard deviation of Ct was below 0.5 among replicates. Results were calculated by the standard curve method.

### 2.8 qPCR absolute telomere length measurement

Telomere length was calculated by means of Absolute Human Telomere Length Quantification qPCR Assay Kit (ScienCell). The kit provided a primer solution for telomere amplification and another one that recognizes and amplifies a 100 base pair region on human chromosome 17. This last primer solution was used as single copy reference (SCR). Twenty nanograms of target DNA was added to a reaction containing the pair of primers (telomere or SCR) and FastStart Essential DNA Green Master (Roche), in a total reaction volume of 20µl, according to the manufacturer's instructions. PCR experiments were carried out on a Stratagene Mx3005P system (Agilent Technologies) and analyzed with MxPro 4.01 QPCR software Stratagene (Agilent Technologies). Telomere and SCR reactions were run in the same 96‐well plate and followed the same qPCR program setup (initial denaturation step at 95ºC for 10 minutes, followed by 32 cycles of 95°C for 20 seconds, 52ºC for 20 seconds and 72°C for 42 seconds, with signal acquisition).

Data were collected from duplicate reactions for each sample (cell lines, patients, and healthy donors). Duplicate values were accepted when the standard deviation of Ct was below 0.5 among replicates. The provided reference genomic DNA sample with known telomere length in kilobases served as reference to calculate samples’ telomere length (2^−∆∆Ct^). The final result represents the average telomere length per chromosome.

### 2.9 Statistical analysis

Statistical analyses were performed on GraphPad Prism (version 5.01) and included the calculation of mean, standard deviation of the mean, and *P* values by paired Mann‐Whitney test (nonparametric t test). Correlations between different telomere length measurement methods were calculated using Pearson's Correlation and *R* ^2^ coefficient of correlation and *P* values were reported. Four independent biological samples were analyzed for each cell line. Data obtained with cells from one sample were considered as one experiment (*n*). The significance level was set as *P* = .05.

## 3 RESULTS

### 3.1 CTCL cells cytogenetic analysis

Cytogenetic investigation consisted of analyzing the karyotype for all cell lines (MyLa, HuT78, Mac1, Mac2A, and Mac2B). Thus, chromosomal rearrangements (Table S1) and ploidy (Table 1) were determined. HuT78 cell line was hypertriploid (77 to 81 chromosomes), all others cell lines were near‐diploid. MyLa had 47 to 49 chromosomes, Mac1 had 45 to 47, Mac2A had 45 to 46, and Mac2B had 44 to 45. Full chromosomal formulas are available in Table S1. For Sz patients, the complex karyotype was determined by mFISH. All Sz patients (1 to 9) were near‐diploid, except patient 10 who was triploid.​

**Table 1:** CTCL cells’ absolute telomere length estimated by absolute qPCR

	Diploid telomere length (kb)	Ploidy	Corrected telomere length (kb)
Cell lines			
Mac1	1.075 ± 0.035	Near‐diploid	1.075 ± 0.035
Mac2A	4.452 ± 0.147	Near‐diploid	4.452 ± 0.147
Mac2B	2.811 ± 0.093	Near‐diploid	2.873 ± 0.095
MyLa	12.592 ± 0.416	Near‐diploid	12.471 ± 0.412
HuT78	1.858 ± 0.061	Hypertriploid	1.279 ± 0.042
Mean			4.320 ± 0.143
Sz patients			
1	2.819 ± 0.093	Near‐diploid	2.819 ± 0.093
2	3.656 ± 0.121	Near‐diploid	3.656 ± 0.121
3	5.559 ± 0.183	Near‐diploid	5.559 ± 0.183
4	5.392 ± 0.178	Near‐diploid	5.392 ± 0.178
5	2.930 ± 0.097	Near‐diploid	2.930 ± 0.097
6	4.623 ± 0.153	Near‐diploid	4.623 ± 0.153
7	2.077 ± 0.069	Near‐diploid	2.077 ± 0.069
8	8.226 ± 0.272	Near‐diploid	7.883 ± 0.260
9	3.387 ± 0.112	Near‐diploid	3.462 ± 0.114
10	3.804 ± 0.126	Triploid	2.536 ± 0.084
Mean			4.094 ± 0.135

## 4 CTCL CELLS TELOMERE LENGTH

### 4.1 Relative and absolute telomere length measurements

**Figure 1:**
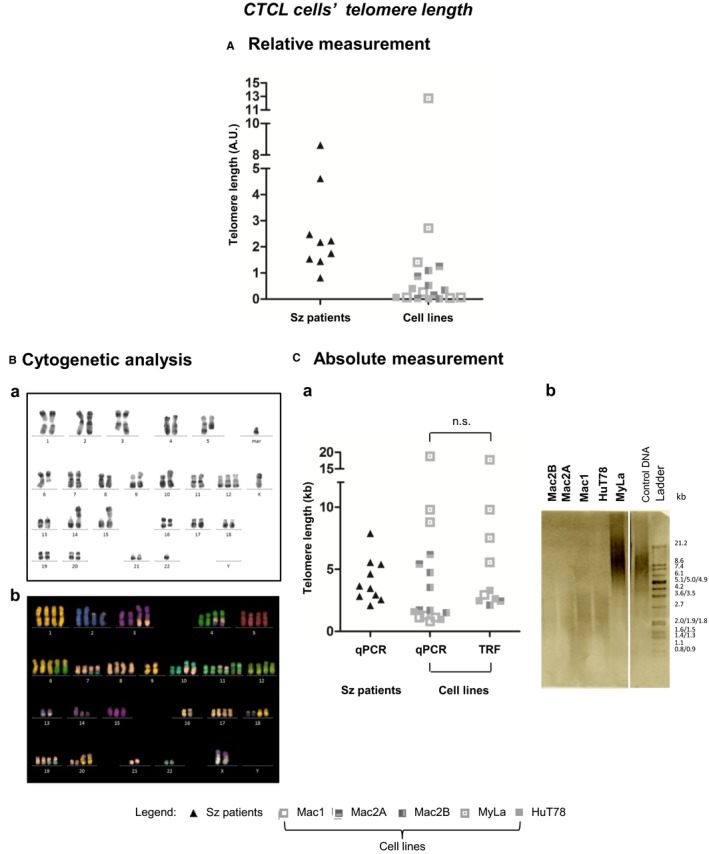
CTCL cells’ telomere length assessment. (A) Relative telomere length measurement by a standard relative qPCR assay. (B) Cytogenetic analysis of CTCL cells (a) Conventional karyotype of a near‐diploid cell and (b) mFISH of a hypertriploid karyotype (C) Absolute telomere length measurement (a) by qPCR and by TRF. The mean cell lines’ telomere length estimated by qPCR (4.320 ± 0.143 kb) was similar to that estimated by TRF (5.652 kb), *P* = .5040. (b) TRF blot. Arbitrary units (AU); Cutaneous T‐cell lymphoma (CTCL); Deoxyribonucleic acid (DNA); Kilobases (kb); Nonstatistically significant (n.s.) Quantitative real‐time polymerase chain reaction (qPCR); Sézary (Sz); Terminal restriction fragment (TRF)

We measured the relative and the absolute telomere length of Sz patients at one point and four independent biological samples for CTCL cell lines (Figure 1).

The relative telomere length was assessed by means of a standard qPCR method (Figure 1A), with a mean variation between measurements (inter‐CV) of 13.6% and an individual sample variation (intra‐CV) of 8.4%. Using this method, we were able to measure the telomere length of 9 (out of 10) Sz patients, since we never succeeded to amplify neither the reference gene nor the telomeres for one patient (Figure 1A). In cell lines the absolute telomere length was assessed by qPCR (inter‐CV of 6.7% and intra‐CV of 2.5%) and by measuring the TRF length means (inter‐CV of 6.3%) (Figure 1C). These two methodologies were applied only on cell lines due to the huge amounts of DNA required for TRF analysis, which was a limitating factor for analyzing Sz patients.

qPCR absolute telomere lengths were calculated considering cell ploidy: the average telomere length per chromosome was calculated by dividing the cell average telomere length over the number of chromosomes per cell (Table 1). With this absolute qPCR method we succeeded to calculate the telomere length for all Sz patients (Figure 1Ca). Obtained results, using different telomere length measurement methods, were concordant and allowed us to conclude that Mac1 and HuT78 presented the shortest telomeres, with stable telomere length variation between independent biological samples (Figure 1A and Figure 1Ca). Mac2A and Mac2B presented longer telomeres than HuT78 and Mac1, as well as more variability in their telomere length (Figure 1A and Figure 1Ca). MyLa was the cell line with the longest telomeres among all the cell lines we studied, as well as the one with the highest variability in their telomere length measurement (Figure 1A and Figure 1Ca). The mean cell lines’ telomere length estimated by qPCR (4.320 ± 0.143 kb) was similar to that estimated by TRF (5.652 kb), *P* = .5040 (Figure 1Ca).

**Figure 2:**
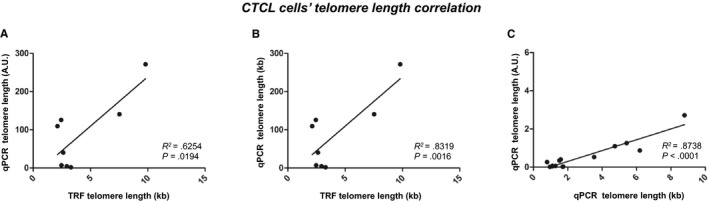
CTCL cells’ telomere length assays correlation. Telomere length results estimated by TRF correlated with results from relative qPCR (A) and with results from absolute qPCR (B). Telomere length estimation by qPCR‐based assays correlated with each other (C). Arbitrary units (AU); Correlation coefficient (R^2^); Cutaneous T‐cell lymphoma (CTCL); Kilobases (kb); Quantitative real‐time polymerase chain reaction (qPCR); Terminal restriction fragment (TRF)

Telomere length results estimated by TRF correlated with results from relative (Figure 2A) and absolute (Figure 2B) qPCR approaches (*R^2^* = 0.6254, *P = *.0194 and *R^2^* = 0.8319, *P = *.0016, respectively). Telomere length estimation by qPCR‐based assays (Figure 2C), strongly correlated with each other (*R^2^* = 0.8738, *P* < .0001).

### 4.2 DNA sample quality

**Figure 3:**
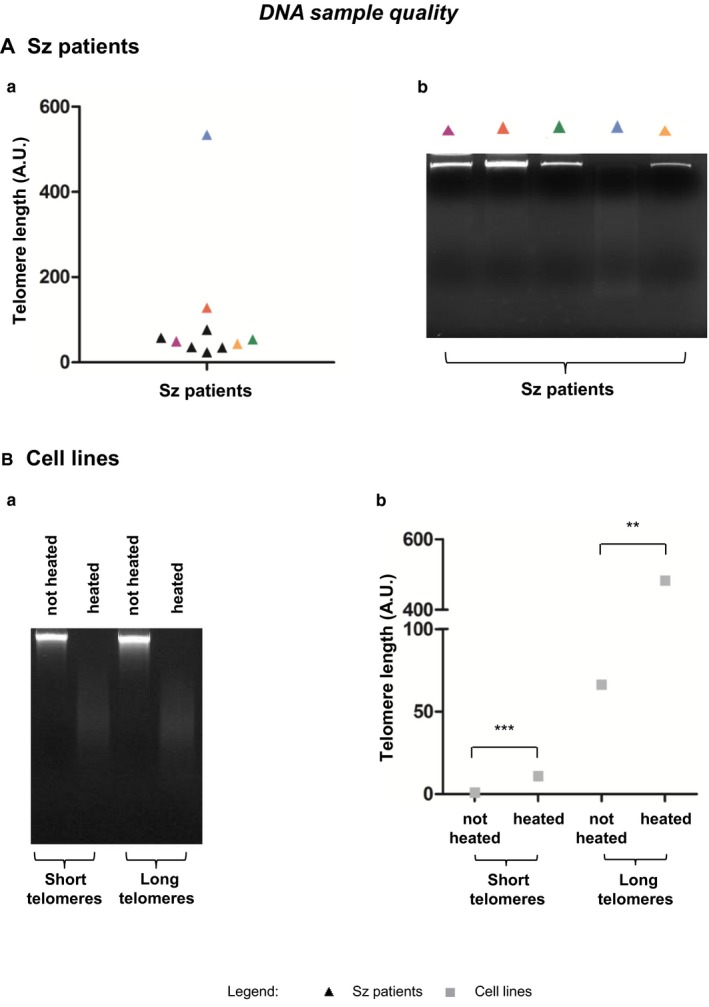
Influence of DNA quality on telomere length measurement. (A) Sézary (Sz) patients’ (a) relative qPCR telomere length measurement and (b) patient samples marked in colored triangles DNA quality analysis by agarose gel electrophoresis. (B) Two cell lines (one with short telomeres and another with long telomeres) (a) DNA heat degradation confirmed by agarose gel electrophoresis and (b) their relative qPCR telomere length measurement. Telomere length of both cell lines significantly increased following DNA degradation (*P* = .0001 for short telomere cell line and *P* = .0037 for long telomere cell line). Arbitrary units (AU); Sézary (Sz); **P < .01; ***P < .001

When analyzing Sz patients’ telomere length, we observed the occurrence of an “outlier” far from patients’ average telomere length (Figure 3Aa). We verified samples' quality by agarose gel electrophoresis and we found that it was due to DNA degradation (Figure 3Ab). Thus, this patient was excluded from this study. This was further investigated in two cell lines, one with short telomeres and another one with long telomeres (Figure 3B). When DNA was degraded by heating (Figure 3Ba), the telomere lengths significantly increased (Figure 3Bb). We compared the *KLK3* (reference gene) and telomeres Ct values of both cell lines. We observed that the most remarkable difference between undegraded and degraded DNA was at the level of *KLK3* gene Ct. Indeed, *KLK3* gene Ct value increased in degraded DNA (Table 2).

**Table 2:** Ct values for KLK3 and Telomeres of two cell lines following heat degradation

Cell lines		Ct (KLK3)	Ct average	Ct (Telomeres)	Ct average	2^(‐ΔCt)^
Short telomere	not heated	24.00	23.95	24.05	24.09	0.90
		23.89		24.13		
	heated	26.87	26.83	23.38	23.40	10.82
		26.79		23.41		
Long telomere	not heated	22.07	22.14	16.18	16.09	66.16
		22.20		16		
	heated	24.06	24.17	15.33	15.26	483.36
		24.28		15.18		

### 4.3 Sample’ tumor cell percentage

**Figure 4:**
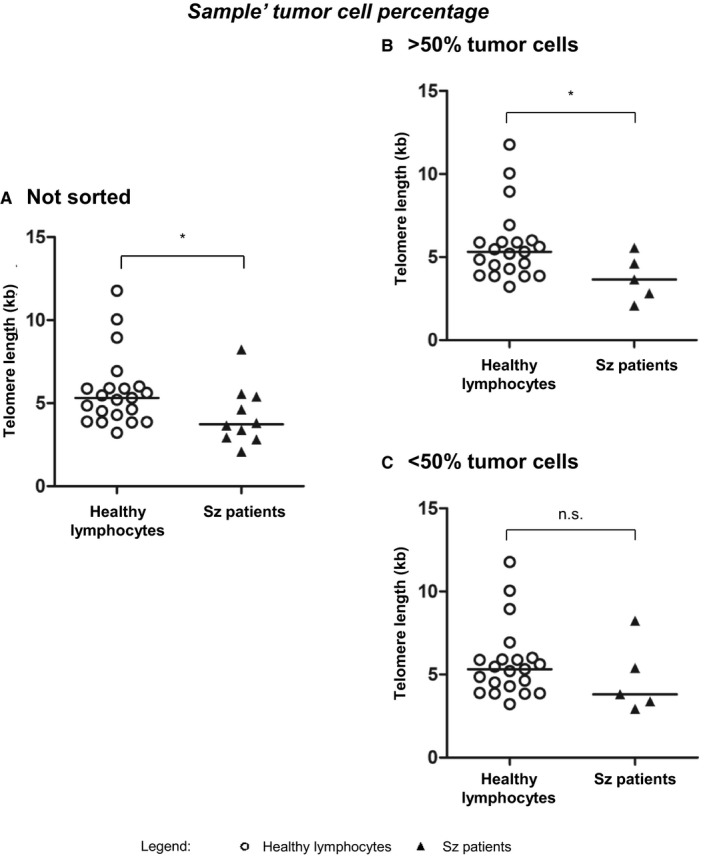
Influence of samples’ tumor cell percentage on telomere length in comparison with healthy donors. (A.) Telomere length of Sz patients’ samples not sorted were significantly shorter when compared with that of healthy lymphocytes (*P* = .0238). (B.) Telomere length of Sz patients’ samples with more than 50% of tumor cells have significantly shorter telomeres that those of healthy lymphocytes (*P* = .0374). (C.) Telomere lengths of Sz patients’ samples with less than 50% of tumor cells were not statistically different from those of lymphocytes from healthy donors (*P* = .1719). Kilobases (kb); Nonstatistically significant (n.s.); Sézary (Sz); *P < .05

We observed that the telomere length of our Sz patient cohort (Figure 4A) was significantly shorter when compared with that of healthy lymphocytes (*P* = .0238). We then compared the telomere lengths based on samples’ tumor cell percentage (Figure 4B,C). We observed that samples with more than 50% of tumor cells (Figure 4B) had significantly shorter telomeres than those of healthy lymphocytes (*P* = .0374), while telomere lengths of samples with less than 50% of tumor cells (Figure 4C) were not statistically different from those of lymphocytes from healthy donors (*P* = .1719).

## 5 DISCUSSION

In the present study we intended to evaluate and compare methods to ascertain telomere length in clinical samples using as a model Sézary syndrome disease, an aggressive CTCL subtype. We also aimed to identify putative factors interfering with an accurate evaluation.

We used a qPCR commercial kit to measure the absolute telomere length of CTCL cells. As a commercial kit, it is assured to render results with high reliability, sensitivity, and reproducibility, and to reduce intra and interassays discrepancies.^37^ Furthermore, it allows obtaining telomere length in absolute kilobases, otherwise only possible by TRF analysis. TRF, although considered as the “gold standard” for telomere length evaluation, requires large DNA quantities which constraints its applicability to cancer study. We often do not have access to large amount of cells or genetic material, so qPCR presents an advantageous tool.^27^.

The main hurdle in using qPCR‐based techniques to explore cancer cells relies on the selection of an appropriate reference gene.^27^, ^29^ Ploidy abnormalities and chromosome rearrangements are commonly associated with cancer development, making it very likely to select a reference gene that is amplified or lost.^38^ Cytogenetic data allowed us to investigate chromosome 17 status of CTCL cells, and this information was important since the qPCR kit uses a 100 base pair‐long region on this chromosome as a reference. By cytogenetic data, we guaranteed (under the resolution limit of around 5MB), the selection of a stable reference gene for qPCR relative telomere length measurement, and we verified that the single copy gene reference proposed by the qPCR kit is suitable for CTCL absolute telomere length measurement. Karyotype information was furthermore essential to complement the advantages of telomere qPCR, as cell ploidy allowed the correct calculation of the average telomere length per chromosome (Table 1). This is particularly important because when studying cancer cells, the single telomere length measurement by itself has no biological meaning if not compared to the telomere length of a representative healthy population. Hence, the correct telomere length calculation is extremely important to assess and discover associations between telomere length and a certain disease. In this work, the majority of CTCL cell lines and Sz patients were near‐diploid, so the ploidy did not influence telomere length result. However, for HuT78 cell line and patient 10 that presented a near‐triploid and a triploid karyotype, respectively, the ploidy correction factor influenced telomere length measurement (Table 1).

Regarding telomere length results obtained with the different measurement methods (Figure 1), the qPCR‐based results, which specifically measures telomere sequences, are concordant with each other (Figure 2C). TRF analysis, on the other hand, measures the telomeres including their subtelomeric region, which generally overestimates telomere length of around 1kb.^18^ Indeed, mean cell lines’ telomere length estimated by TRF (5.652 kb) is around 1kb greater than that estimated by qPCR (4.320 ± 0.143 kb) (Figure 1Ca).

Another crucial aspect of telomere length measurement is DNA quality. It is established that one of the primary requests for qPCR‐based techniques in general, and for telomere qPCR in particular, is the use of DNA of high quality.^29^ Indeed, we verified that DNA degradation strongly influences telomere length measurements (Figure 3). Upon DNA degradation, we observed that the most remarkable difference, between uncompromised DNA and degraded DNA, occurred at the level of KLK3 gene Ct (our reference gene) (Table 2). The number of cycles to obtain a detectable log‐linear phase of amplification increased upon DNA degradation, which means that we obtained less KLK3 product amplification in degraded samples. Consequently, as the telomere amplification did not significantly change, the ratio telomere/KLK3 decreased and this translated into longer telomeres (Table 2 and Figure 3Bb). This is in contradiction with TRF method, where DNA degradation produces a bias toward shorter lengths.^19^ Thus, we emphasized the importance of regularly check samples’ DNA quality.

Finally, we reinforced the impact of analyzing samples with high percentage of tumor cells, as it can influence telomere length evaluation relatively to healthy lymphocytes (Figure 4). On one hand, samples with more than 50% of tumor cells presented significantly shorter telomere lengths, compared to healthy lymphocytes. On the other hand, samples with less than 50% of tumor cells presented telomeres with no statistical difference from healthy lymphocytes. This corroborated our previous observations that short telomere length is a characteristic of Sz tumor cells and that the surrounding nontumor cells present longer telomeres.^30^ Therefore, the analysis of samples with high tumor cell proportion will grant more precise results providing a way to accurately distinguish unhealthy from healthy population. We further assured that the telomere length of Sz patients was not due to their advanced ages (Figure S1). Hence, we discriminated between natural telomere shortening and a pathological decrease, which is a hallmark of Sz cells.^30^

In conclusion, the increased utility of telomere length assessment as a biomarker of cancer cells emphasized the importance of accurate telomere length estimation.

Cancer cells accumulate genetic and chromosomal abnormalities and we do not always have access to a large amount of cells or genetic material to work with. The qPCR‐based techniques used to assess telomere length can overcome these problems. Our results, limited by being performed in an uncommon disease which did not allow statistical power calculation, indicate that accurate measurements can only be obtained, with high tumor cell representation samples, undegraded DNA, well‐defined cell ploidy, and a known chromosomal status.

## CONFLICT OF INTEREST

The authors declare no competing financial interests.

## AUTHOR CONTRIBUTIONS

Joana Ropio and Alain Chebly wrote the original draft, Paula Soares, Jean‐Philippe Merlio, and Edith Chevret edited. All authors provided substantial contributions, conception and design, acquisition of data, or data analysis and interpretation. Edith Chevret supervised all the research. All authors gave final approval of the submitted version.
