# Supplementary material for: Reliable blood cancer cells' telomere length evaluation by qPCR
Source: Cancer Med. 2020 Mar 6;9(9):3153–62. doi: 10.1002/cam4.2816 (PMC7196062; doi:10.1002/cam4.2816)
Supplement: Supplementary file 1 [file CAM4-9-3153-s001.pdf]

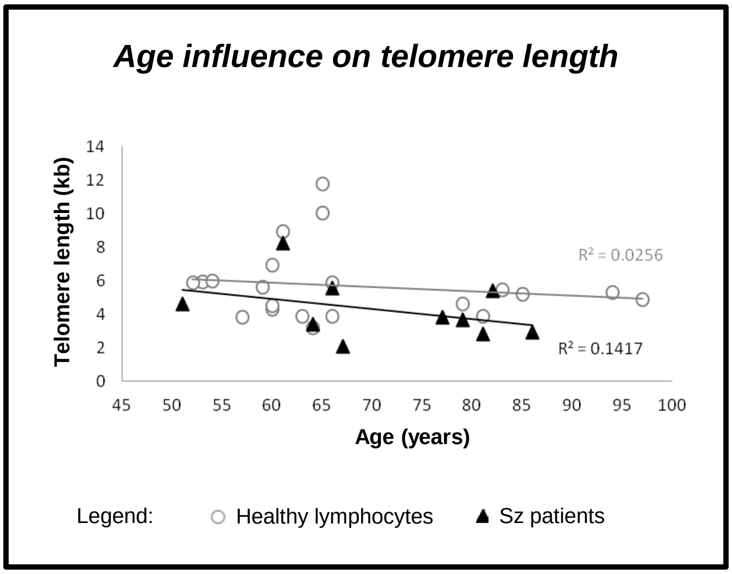

Supplementary Figure 1.
