# Supplementary material for: Reliable blood cancer cells' telomere length evaluation by qPCR
Source: Cancer Med. 2020 Mar 6;9(9):3153–62. doi: 10.1002/cam4.2816 (PMC7196062; doi:10.1002/cam4.2816)
Supplement: Supplementary file 2 [file CAM4-9-3153-s002.docx]

**Supplementary table 1 - CTCL cell lines chromosomal formulas**

| **Cell line** | **Chromosomal formula** *according to ISCN 2016* |
| --- | --- |
| **MyLa** | 47~49,XY,del(1)(q32)[10],der(1)t(1;15)(p36;q15)[10],del(2)(p?16)[8], der(2)t(2;14)(q32;q12)[10],t(4;5)(q13;q15)[10], der(6)?add(6)(p22)del(6)(q15;q23)[10],del(7)(p15)[10],+del(7)(p1?5)[10], del(9)(p21)[9],der(10)t(10;13)(p14;q13)[10],der(13)t(13;16)(q14;?q23)[10], der(14)(2pter🡪2p13::14p12🡪14q12::2q32🡪2qter)[10],der(15)t(1;15)(q32;q15)[10],  +17[10],der(18)t(18;?19)(q23;?p13)[10],der(19)t(?11;19)(?;p13)[10],  der(20)t(20;?)(q13;?)[10],der(21)t(?16;21)(?;q22)[10],+der(?)(?acro)[10][cp10] |
| **HuT78** | 77~81<3n>,Y,t(X;13)(p11;q14)x2[5],+?der(2;20)(p10;p10)[5],+3[5] ,  der(3)t(3;10)(q29;q24)x2[5],+?4[5],+?4[5],  der(4)(4pter🡪4p?14::4?q21🡪4?p14::4?q25🡪4?q21::16p?11🡪16p?13::13q14🡪13qter)x2[5],+?5[3],t(5;6)?(p10;p10)x2[5],+der(6)t(4;6)(q2?1;p?21)x2[5],  der(7)t(7;10)(p14;?q11)x2[5],-9[5],der(10)t(7;10)(q11;q22)[4],del(10)(q24)[5],  ?del(11)(q?)[5],?del(12)(p13)[4],-13[4],+14[5],del(14)(q?11q?24)x2[5],+15[4],  16[5],+17[5],der(18)t(2;18)(p22;p11)x2[5],+der(19)t(19;20)(q13;q11)[5], der(19)t(19;20)(q13;q11)[5],der(19)t(19;22)(q10;q10)x2[5],+der(20)t(19;20)(?;p11)[4], del(20)(?q11q13)[3],der(20)(:9p11🡪9p24::20p13🡪20qter)x2[5],  der(21)t(11;21)(q?13;q10)[5][cp5] |
| **Mac1** | 45~47,XY,der(4)?t(4;5)(p16;?)[7],t(8;9)(p22;p2?4)[7],-13[5], der(14)?(:9::13q?32🡪13p10::14p?10🡪14qter)[7],-18[7],+21[7][cp7] |
| **Mac2A** | 45~46,X,-Y[10],?dup(3)(?q25q28)[10],del(5)(?q12q25),  del(6)(q15)[10],t(8;9)(p22;p2?4)[10],der(10)t(6;10)(q15;p11)[9],+12[5],  del(12)(q11q13)[10],?i(14)(q10)[9],der(15)t(2;15)(p11;p12)[10], del(16)(q11q22)[10],inv(20)(?p13q13)[10],-22[10],+der(?)(?acro)[10][cp10] |
| **Mac2B** | 44~45,X,-Y[10],del(6)(q15)[10],der(8)t(8;9)(p22;p2?4)[10],  der(9)t(8;9)(p22;p2?4)?inv(9)[10],der(10)t(6;10)(q15;p11)[10],del(12)(q11q13)[10],  dup(12)(q12q13)[10],der(15)t(2;15)(p11;p12)[10],  der(21)t(21;?)(p11;?)[9],der(22)t(22;14)(p11;?)[7],+der(?)[4][cp10] |
